# Effectiveness of Non-Pharmacological Interventions for Sleep Disorders in Enhancing Quality of Life, Cognitive Function, and Sleep Quality in Older Adults with Mild Cognitive Impairment: A Systematic Review and Meta-Analysis

**DOI:** 10.3390/medicina61040583

**Published:** 2025-03-25

**Authors:** Edgar Vásquez-Carrasco, Maria Rojas, Lukas Larenas, Aline Ferrada, Jordan Hernandez-Martinez, Francisco Ahumada-Méndez, Marcelo Leiva-Bianchi, Florencia Carmine, Cristian Sandoval, Braulio Henrique Magnani Branco, Pablo Valdés-Badilla

**Affiliations:** 1 School of Occupational Therapy, Faculty of Psychology, Universidad de Talca, Talca 3465548, Chile; edgar.vasquez@utalca.cl (E.V.-C.); mariaemiliarojas95@gmail.com (M.R.); larenas.lukas29@gmail.com (L.L.); b.f.alineflorencia@gmail.com (A.F.); 2 Centro de Investigación en Ciencias Cognitivas, Faculty of Psychology, Universidad de Talca, Talca 3465548, Chile; 3 Department of Physical Activity Sciences, Universidad de Los Lagos, Osorno 5290000, Chile; jordan.hernandez@ulagos.cl; 4 G-IDyAF Research Group, Department of Physical Activity Sciences, Universidad de Los Lagos, Osorno 5290000, Chile; 5 Programa de Investigación en Deporte, Sociedad y Buen Vivir, Universidad de los Lagos, Osorno 5290000, Chile; 6 Laboratory of Methodology, Behavior and Neuroscience, Faculty of Psychology, Universidad de Talca, Talca 3465548, Chile; francisco.ahumada@utalca.cl (F.A.-M.); marcleiva@utalca.cl (M.L.-B.); 7 Carrera de Medicina, Facultad de Medicina, Universidad de La Frontera, Temuco 4811230, Chile; f.carmine02@ufromail.cl; 8 Escuela de Tecnología Médica, Facultad de Salud, Universidad Santo Tomás, Los Carreras 753, Osorno 5310431, Chile; 9 Departamento de Medicina Interna, Facultad de Medicina, Universidad de La Frontera, Temuco 4811230, Chile; 10 Núcleo Científico y Tecnológico en Biorecursos (BIOREN), Universidad de La Frontera, Temuco 4811230, Chile; 11 Graduate Program in Health Promotion, Cesumar University (UniCesumar), Maringá 87050-900, Brazil; braulio.branco@unicesumar.edu.br; 12 Department of Physical Activity Sciences, Faculty of Education Sciences, Universidad Católica del Maule, Talca 3530000, Chile; 13 Sports Coach Career, School of Education, Universidad Viña del Mar, Viña del Mar 2520000, Chile

**Keywords:** aged, cognitive function, cognitive impairment, older adults, quality of life, sleep disorders, sleep quality

## Abstract

*Background and Objectives*: This systematic review with meta-analysis aimed to evaluate and synthesize the scientific evidence of interventions for sleep disorders on sleep quality, cognitive function, and quality of life in older adults with mild cognitive impairment (MCI). *Materials and Methods:* A systematic literature search was conducted between April 2024 and January 2025 using the Core Collection of seven generic databases: PubMed, EBSCOhost, CINAHL Complete, Cochrane, Scopus, ProQuest, and Web of Science. The PRISMA, RoB 2.0, and GRADEpro tools assessed the evidence’s methodological quality and certainty. The protocol was registered in PROSPERO (CRD42024558406). *Results*: A total of 2815 records were found in the databases, of which eight studies were analyzed using the PICOS format. There was a significant large effect (*p* = 0.005) in favor of the experimental group compared to the control group in the Sleep Quality Index (*p* = 0.005). No significant differences were reported for the other variables studied. *Conclusions*: Interventions for sleep disorders in older people with MCI aimed at improving sleep quality demonstrated significant effects assessed with PSQI. Individual results demonstrated limited effects on cognitive function and quality-of-life assessments.

## 1. Introduction

The aging process is associated with multiple changes, including alterations in the sensory system, reduced muscle strength, loss of mobility, increased susceptibility to chronic diseases, neurological disorders, and cognitive decline [1]. Similarly, older adults often experience changes in sleep quality, particularly in cases of pathological aging, with studies indicating that poor sleep quality negatively impacts cognitive functions [2]. A common issue among older adults is mild cognitive impairment (MCI), which is marked by challenges in learning and memory, as well as difficulty concentrating on tasks for both short and long durations [3]. This neurodegenerative process leads to a reduction in the connectivity of neural networks [4]. MCI is common in older people, and its prevalence increases with the age and educational level of the person. Furthermore, older people diagnosed with MCI may remain stable or progress to dementia [5]. A systematic review conducted by Pais et al. [6] revealed that the global incidence of MCI is 54 per 1000 person-years. Similarly, a meta-analysis showed the average global prevalence of MCI, 15.6% [7]. In addition to the above, approximately 46% of older people with MCI progress to dementia within 3 years [8]. Various studies have shown that poor sleep quality affects cognitive functions because sleep’s primary function is to restore the nervous system [2,9]. About the above, 20% to 40% of older people who present cognitive alterations may develop sleep disorders, such as insomnia, obstructive sleep apnea, and restless legs syndrome, among others [10]. Sleep disorders can be linked to decreased quality and quantity of sleep [2]. Likewise, they can be defined as any alteration of the sleep process, including difficulties such as falling asleep, getting up during the night, waking up early, and experiencing excessive daytime sleepiness [11]. These have become a common condition and are increasing over the years, especially in older people [12], and may have adverse effects on cardiovascular diseases, neurocognitive functions, psychological disorders, metabolic abnormalities, and immune response [13]. Sleep disorders are associated with neurocognitive alterations, attentional difficulties, and impaired cognitive performance, which influence daytime functioning, as well as the social and work context of the person, generating a poor quality of life [14]. Bubu et al. [15] revealed in a general meta-analysis that individuals with sleep disorders face a 1.68 times higher risk of developing cognitive impairment. Poor sleep quality has important implications for health, as it can hurt physical and mental health, interfering with daily functioning [16]. These sleep disorders reduce older people’s quality of life and are related to defective functioning in various areas, including memory and executive functions [17]. Cognitive impairment causes a decrease in the user’s quality of life, where any intervention that manages to mitigate cognitive symptoms could reduce disability and improve the quality of life of these older people [18]. One of these interventions may be cognitive stimulation, which can promote neuronal plasticity and the development of the brain’s compensatory networks and thus favor cognitive function [19]. There is also pharmacology, where the vast majority of drugs have not been successful in clinical trials, obtaining as results postponing the development of cognitive impairment instead of improving or slowing it [20].

Various studies on improving sleep quality throughout history have focused mainly on pharmacological interventions, but disadvantages can be observed regarding the side effects that this produces [21]. Non-pharmacological interventions can improve sleep quality, increase its duration, and prevent the onset of sleep disorders [22]. Some of the current interventions used in sleep disorders are, for example, cognitive behavioral therapy (CBT), which aims to change cognitive dysfunctions and maladaptive behaviors [23]. On the other hand, alternatives such as physical activity interventions have shown favorable results in improving sleep quality [24], where physical activity can prevent complications in sleep quality and can be used as a complementary technique to cognitive behavioral therapy, which is the first-line intervention in sleep disorders [25]. Therefore, this systematic review with meta-analysis aimed to evaluate and synthesize the scientific evidence of interventions for sleep disorders on quality of life, cognitive function, and sleep quality in older people with MCI.

## 2. Methods

### 2.1. Protocol and Registration

This systematic review with meta-analysis followed the Cochrane Collaboration methodology [26] and adheres to the PRISMA checklist and flowchart standards for reporting [27]. The PROSPERO database (University of York, York, UK) registered the review protocol with the identification code CRD42024558406.

### 2.2. Eligibility Criteria

This systematic review with meta-analysis included peer-reviewed original articles, specifically randomized controlled trials (RCTs), with no language or publication date restrictions until January 2025. Excluded materials were conference abstracts, books and book chapters, editorials, letters to the editor, protocol records, reviews, case studies, and non-randomized trials. The PICOS (Population, Intervention, Comparator, Outcome, Study Design) framework was used to guide the inclusion of studies (Table 1).

### 2.3. Information and Database Search Process

Seven databases were consulted: PubMed (National Center for Biotechnology Information), Web of Science (Core Collection), Scopus, EBSCOhost, ProQuest, Cochrane Library, and CINAHL Complete. The Medical Subject Headings (MeSH) of the National Library of Medicine of the United States of America and free phrases related to cognitive impairment, sleep quality, quality of life, cognitive function, and older people were used. The following were used: (“Sleep apnea syndromes” OR “Sleep initiation and maintenance disorders” OR “Restless legs syndrome” OR “Narcolepsy” OR “Parasomnias” OR “Sleep bruxism” OR “Sleep-wake disorders” OR “Sleep disorders, intrinsic” OR “Dyssomnias” OR “Sleep deprivation” OR “REM sleep parasomnias” OR “Sleep fragmentation” OR “Sleep insufficiency” OR “Inadequate sleep” OR “Sleep debt” OR “Insomnia” OR “Nightmare disorder”) AND (“Quality of life” OR “Lifestyle” OR “Healthy lifestyle” OR “Self-care” OR “Functional status” OR “Living conditions” OR “Lifestyle factor”) AND (“Cognitive dysfunction” OR “Neurocognitive disorders” OR “Cognitive disorder” OR “Cognitive impairment” OR “Mild cognitive impairment” OR “Cognitive decline” OR “Mild cognitive changes” OR “Cognitive decline syndrome” OR “Early cognitive decline” OR “Minor cognitive decline”) AND (“Cognitive training” OR “Electroencephalography” OR “Psycho-motor performance” OR “Neuropsychological testing” OR “Cognition” OR “Executive function” OR “Brain function” OR “Cognitive process” OR “Cognitive processes” OR “Cognitive processing” OR “Cognitive performance” OR “Cognitive function” OR “Cognitive functions”) AND (“Elderly” OR “Aged” OR “Elderly person” OR “Elderly adult” OR “Older people” OR “Older adults” OR “Aging” OR “Older subject”).

An independent expert was consulted about the included articles and the inclusion and exclusion criteria to help find more relevant studies. We established two requirements that the expert had to meet: (i) hold a PhD in health sciences and (ii) have peer-reviewed works published in journals with an impact factor, according to Journal Citation Reports^®^, on cognitive decline, sleep quality, cognitive function and quality of life in adult population and/or older people. Our search approach was not revealed to the expert to avoid bias in his searches. After completing these procedures, in January 2025, we searched the database to find relevant retractions or errata related to the included articles.

### 2.4. Study Selection and Data Collection Process

Studies were exported to Mendeley Reference Manager (version 2.116.1, Mendeley Ltd., London, UK), and the study selection process is illustrated in the PRISMA flowchart. Three authors (A.F., L.L., and M.R.) independently performed the searches, systematically reviewing titles, abstracts, and full texts and removing duplicates. At this stage, no discrepancies were identified. Potentially eligible articles were subsequently thoroughly re-screened, and the exclusions of those not meeting the predefined selection criteria were justified. Finally, an additional reviewer (E.V.-C.) independently audited the entire selection and data extraction process.

### 2.5. Methodological Quality Assessment

The studies’ level of evidence and methodological quality were evaluated based on the Oxford Center for Evidence-Based Medicine scale (University of Oxford, Oxford, UK) for clinical oncology. Only level 1a studies, defined as RCTs, were included. Studies classified as levels of evidence 1b, 2a, 2b, 3a, 3b, 4, and 5 were excluded. RCTs were downgraded if concerns were raised regarding the risk of bias, consistency, accuracy, precision, transparency of results, or publication bias [28].

### 2.6. Data Collection Process

Relevant data were extracted from each study included in the systematic review with meta-analysis and recorded using a data extraction form, following Cochrane recommendations [26], with Microsoft Excel^®^ software (version 16.81, Microsoft Corporation, Redmond, WA, USA). The data extraction was conducted independently by three authors (A.F., L.L., and M.R.), who subsequently compared the results of their analyses. The entire extraction process was jointly overseen by (E.V.-C.). The extracted variables from each study included title, author/year, country of origin, level of evidence, study design, risk of bias, population and sample size, inclusion criteria, study setting, intervention and control groups (CG), outcome measures, and results.

### 2.7. Risk of Bias in Individual Studies

The risk of bias in individual studies was assessed using the Cochrane Risk of Bias Tool for Randomized Controlled Trials (RoB 2.0, Cochrane Collaboration, London, UK) across its 5 domains (Randomization Bias, Intervention Bias, Missing Outcome Data, Outcome Measurement, and Selection of Reported Outcome) [29]. Three authors (A.F., L.L., and M.R.) jointly completed the RoB analysis, which was reviewed by another author (E.V.-C.).

### 2.8. Measures for Meta-Analysis

The study methodology incorporated a meta-analysis, with full details available on PROSPERO (CRD42024558406). The standardized mean difference (SMD), a statistic that measures the absolute difference between mean values in two groups within an RCT, was calculated for each analysis using Comprehensive Meta-analysis Software (RevMan 5.4, The Cochrane Collaboration, London, UK). A *p*-value of <0.05 was considered statistically significant [30]. For each trial, a random-effects model, based on the Der Simonian-Laird approach, was employed to calculate and pool the SMD and mean difference (MD) in quality of life, cognitive function, and sleep quality from pre- to post-intervention, comparing experimental and CG [31]. The underlying assumption of the random-effects model is that true effects, such as intervention type or duration, vary across studies, with data drawn from populations with differing effect sizes. Data were pooled if consistent results were obtained from at least three studies [32]. Heterogeneity among trial results was assessed using the Cochrane Q test [33] and the I^2^ statistic, where I^2^ values of <25%, 25–50%, and >50% represent small, moderate, and substantial inconsistency, respectively [31]. Egger’s regression tests were also conducted to detect small study effects and potential publication bias [34].

### 2.9. Certainty of Evidence

The certainty of evidence from included studies was assessed using the GRADEpro (Grading of Recommendations, Assessment, Development, and Evaluation) framework [35]. Evidence was categorized as high, moderate, low, or very low. All analyses initially started with high certainty, given the inclusion of RCTs, but were downgraded if concerns arose regarding the risk of bias, consistency, accuracy, precision, transparency of results, or publication bias. Three authors (A.F., L.L., and M.R.) independently evaluated the studies, and any discrepancies were resolved by consensus with a fourth author (E.V.-C.).

## 3. Results

### 3.1. Selection of Studies

**Figure 1:**
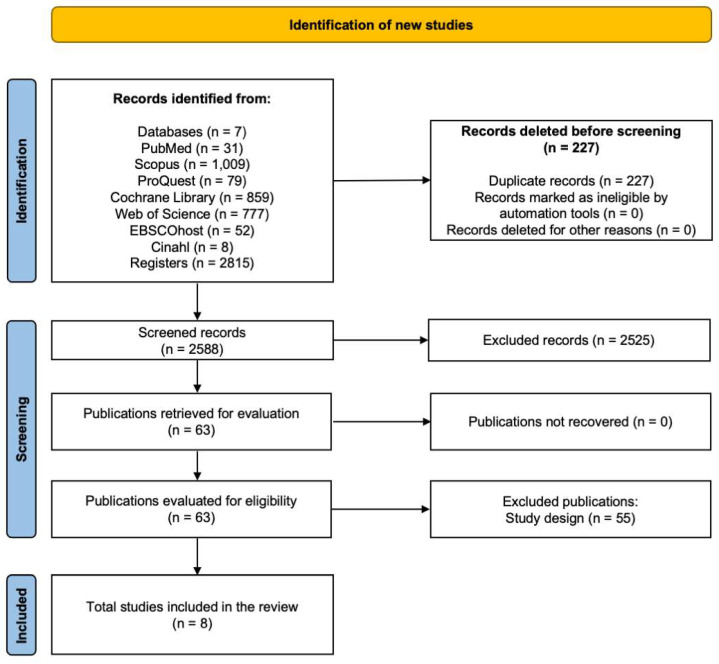
Flowchart of the systematic review.

A total of 2815 studies were identified in all databases (PubMed, Scopus, ProQuest, Cochrane Library, Web of Science, Ebscohost, CINAHL), where 227 studies were excluded due to duplication, of the 2588 articles remaining for evaluation, 2525 were excluded because they did not meet the eligibility criteria after reviewing the titles and abstracts, leaving 63 articles. Subsequently, by reading the full text of the previously selected articles, 55 articles were excluded for not meeting the established inclusion criteria, 28 for including incomplete approaches, 8 for addressing unrelated topics, and 19 for not being RCTs specifically the reason for the study design, leaving a total of eight studies. The search results described are presented by the flowchart (Figure 1).

### 3.2. Methodological Quality

The quality of the studies included in this systematic review with meta-analysis is high because all studies are RCTs, which gives them a high level of evidence according to the Oxford Scale, all reaching level 1a. These types of designs reduce the risk of bias and allow a more precise assessment of the impact of interventions on quality of life, cognitive function and sleep quality in older people with MCI [36,37,38,39,40,41,42,43].

### 3.3. Risk of Bias in Studies

**Figure 2:**
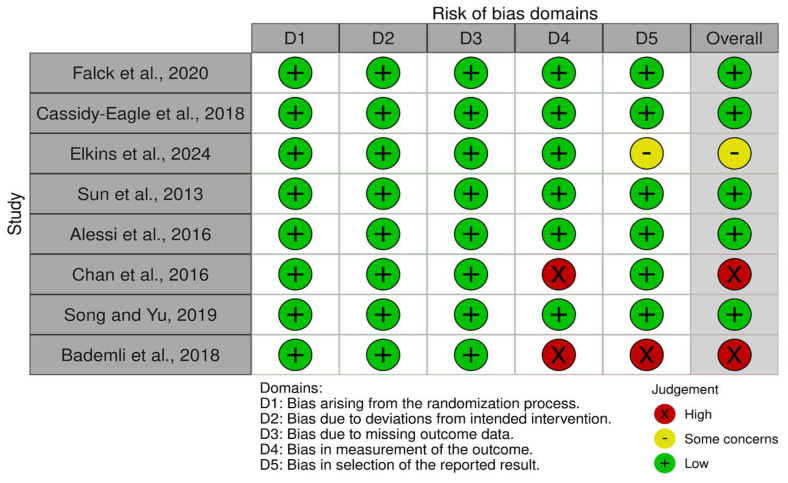
Risk of bias tool: traffic light chart [36,37,38,39,40,41,42,43].

**Figure 3:**
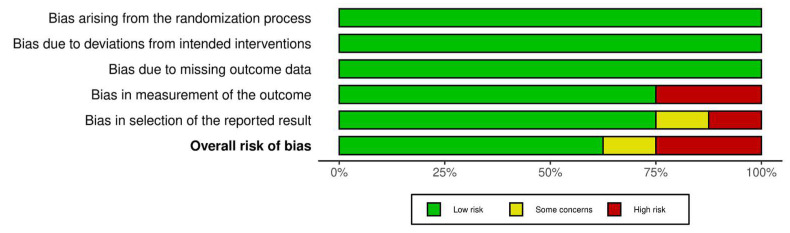
Risk of bias tool: summary table by domain.

Of the studies, five showed a low risk of bias [36,37,39,40,42], one study had some concerns [38], and two studies had a high risk of bias [41,43]. This suggests that the research has a low risk of bias. Figure 2 and Figure 3 summarize the risk of bias in the studies.

### 3.4. Characteristics of the Studies

Of the eight studies analyzed, a favorable impact was evidenced in different interventions related to quality of life, cognitive functions, and sleep quality in older people with MCI. Four studies concluded that cognitive behavioral therapy and physical exercise can significantly improve cognitive function and sleep quality [37,40,42,43]. These studies reinforce the importance of incorporating this type of intervention to improve the quality of life and, on the other hand, the cognitive function of older people. Concerning the central interventions, the eight studies were divided into four categories: (i) multimodal intervention [36]; (ii) cognitive behavioral therapy for insomnia [37,40]; (iii) physical activity program [41,42,43]; and (iv) interventions focused on cognitive stimulation and sleep management [38,39]. The summary of the characteristics of each study and its main results are described in Table 2.

### 3.5. Sample Characteristics

The total population of older people with MCI in this systematic review with meta-analysis was 758 individuals (54.6% female), and the mean age was 74.14 years. The sample size ranged from a minimum of 100 to 150. Twenty-three participants [38] up to a maximum of 159 [40], considering the different results and the type of intervention applied.

### 3.6. Dosages and Interventions Performed

During the interventions, all programs integrated activities to improve sleep quality as an essential component. The interventions varied in duration and frequency. Falck et al. [36] applied 24 weeks with one weekly session of 120 min of multimodal lifestyle intervention, including sleep hygiene, bright light therapy, and physical activity promotion. Cassidy-Eagle et al. [37] opted for 6 weeks with one weekly session of 60 min each, carrying out a cognitive behavioral intervention for insomnia that includes psychoeducational components, sleep hygiene, relaxation, sleep programming, and cognitive therapy. Elkins et al. [38] used 5 weeks with seven weekly sessions of 15 min, carrying out a self-administered hypnosis intervention that implemented audio recordings designed to induce relaxation. Sun et al. [39] implemented 48 weeks with one weekly session of 90 min, where a self-relaxation training intervention was carried out, including sleep hygiene education, progressive muscle relaxation (PMR), and guided meditation. Alessi et al. [40] used 5 weeks with one weekly session of 60 to 90 min, carrying out a cognitive behavioral therapy intervention for insomnia that implements stimulus control, sleep restriction, cognitive therapy, and sleep hygiene. Chan et al. [41] chose to carry out the intervention in 8 weeks with two weekly sessions of 60 min each, of a Tai Chi Qigong intervention. In contrast, Song and Yu [42] applied 16 weeks with three weekly sessions of 60 min, where a moderate-intensity aerobic exercise training intervention was carried out, implementing warm-up, walking exercises, stationary stretching, and step exercises. Finally, Bademli et al. [43] opted for 20 weeks with four weekly sessions of 80 min of a physical activity intervention that includes warm-up activities, rhythmic exercises, cool-down exercises, and free walks.

### 3.7. Quality of Life

A meta-analysis was planned; however, it was not possible due to the diversity of quality-of-life assessments. Nevertheless, the individual results indicate that four studies [36,37,41,42] specifically assessed the effects on quality of life using validated measurement methods. Falck et al. [36] reported no significant differences in %MVPA at 12 weeks (*p* = 0.776) and 24 weeks (*p* = 0.977) after a multimodal lifestyle intervention. Cassidy-Eagle et al. [37] reported a difference only in the SF-36 physical quality of life score (*p* = 0.11) after a cognitive behavioral therapy intervention. Chan et al. [41] reported no significant differences in the physical component of the SF-12 (*p* > 0.05). However, there was a significant improvement in the mental health component score of the SF-12 at 2 months (*p* < 0.001) after a Tai Chi Qigong intervention. Song and Yu [42] recorded a significant improvement in quality of life through the Quality of Life in Alzheimer’s Disease (*p* < 0.001) after a moderate-intensity aerobic exercise training intervention.

### 3.8. Cognitive Function

A meta-analysis was planned; however, it was not possible due to the diversity of quality-of-life assessments. Nevertheless, the individual results indicate that five studies [36,37,39,41,42] specifically evaluated the effects of cognitive function using validated measurement methods. Falck et al. [36] reported that in both groups, there is no significant difference in cognitive function at 12 weeks (*p* = 0.684) and 24 weeks (*p* = 0.349) after multimodal lifestyle intervention. Cassidy-Eagle et al. [37] reported no significant changes in measures of cognitive function (MOCA and HVLT-R) in both groups (*p* = 0.91 and *p* = 0.62, respectively); however, the intervention group showed significant improvement in executive functioning tasks compared to CG (*p* < 0.08) after a cognitive behavioral therapy intervention. Sun et al. [39] reported significant improvements in cognitive function scores over time (*p* < 0.01) in the intervention group after a self-relaxation training intervention. Chan et al. [41] recorded no significant differences in MMSE score or MIC score (*p* > 0.05) in both groups after a Tai Chi Qigong intervention. Song and Yu [42] reported that in the intervention group, there is a significant improvement in cognitive function (*p* < 0.001) compared with the CG after a moderate-intensity aerobic exercise training intervention.

### 3.9. Sleep Quality

**Figure 4:**
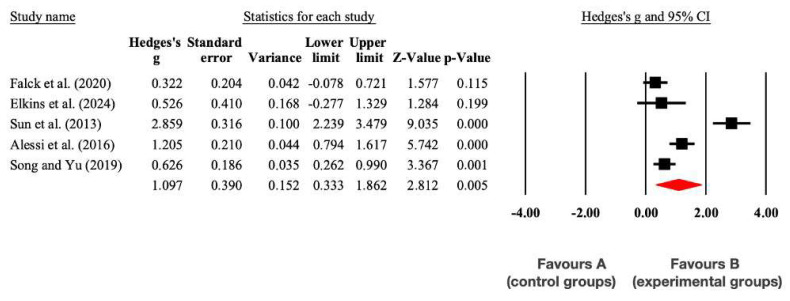
Forest plot of changes in the Sleep Quality Index in older people with MCI participating in non-pharmacological interventions assigned as controls. The shown values are effect sizes (Hedges; g) with 95% confidence intervals (CI). The sizes of the plotted squares reflect the statistical weight of each study. A: control group; B: experimental group [36,38,39,40,42].

There was a significant large effect (*p* = 0.005) in favor of the experimental group (EG) compared to the CG in the Sleep Quality Index (SMD = 1.10; 95% CI = 0.33 to 1.86; I^2^ = 14%; *p* = 0.005). These results are presented in Figure 4.

All studies assessed sleep quality using validated measurement tools [36,37,38,39,40,41,42,43]. Significant improvements were reported in sleep quality, primarily measured by the Pittsburgh Sleep Quality Index (PSQI), following various interventions. Falck et al. [36] observed significant improvements at 24 weeks (*p* = 0.040) after a multimodal lifestyle intervention. Cassidy-Eagle et al. [37] found significant improvements (*p* < 0.001) in sleep outcomes using the Insomnia Severity Index after cognitive behavioral therapy (CBT). Elkins et al. [38] reported significant improvements in the PSQI (*p* < 0.001) and daytime sleepiness on the Epworth Sleepiness Scale (*p* = 0.044) after self-administered hypnosis. Sun et al. [39] observed significant improvements in PSQI scores (*p* < 0.001) following relaxation training. Alessi et al. [40] found significant improvements in PSQI (*p* < 0.05) and Insomnia Severity Index scores (*p* = 0.002) after individual and group CBT, although no significant changes were observed in sleep efficiency via actigraphy (*p* = 0.27). Chan et al. [41] reported improved sleep quality (*p* = 0.004) at 6 months using the Chinese PSQI after a Tai Chi Qigong intervention. Song and Yu [42] observed significant improvements in the PSQI (*p* < 0.001) after moderate-intensity aerobic training. Lastly, Bademli et al. [43] reported significant improvements in the PSQI (*p* < 0.05) following a physical activity program.

### 3.10. Certainty of Evidence

Regarding the results of the certainty of the evidence, these did not allow definitive recommendations to be made on the use of interventions related mainly to multimodal intervention, CBT for insomnia, physical activity programs, and interventions focused on cognitive stimulation and sleep management. Table 3 summarizes the certainty of the studies’ evidence.

### 3.11. Adverse Effects and Adherence

The studies included in this systematic review with meta-analysis reported participant attrition but reported no adverse effects. This indicates that the interventions were well-tolerated and feasible for older adults with MCI, underscoring their potential for broader implementation in similar populations.

## 4. Discussion

### 4.1. Quality of Life

There were no significant differences between EG and CG. A meta-analysis could not be performed due to the heterogeneity of the guidelines used. Within the studies [36,37,41,42], it is evident that, although the multimodal intervention did not significantly improve quality of life, interventions such as CBT, Tai Chi Qigong, and moderate physical activity did have positive effects on various dimensions of quality of life. Different results from RCTs highlight the role of multimodal lifestyle interventions, with significant improvements in quality of life (*p* = 0.0028) observed through the SF-36 assessment, specifically in the general health section [44]. Similarly, a two-arm RCT examining digital CBT for insomnia over 24 weeks reported significant enhancements in sleep-related quality of life (*p* < 0.001) among individuals with insomnia symptoms [45]. Supporting these findings, a meta-analysis of six studies on Tai Chi demonstrated significant improvements in both physical (*p* = 0.007) and mental (*p* = 0.02) quality of life, highlighting its positive impact [46]. Additionally, other studies indicate that aerobic physical activity is a practical therapeutic approach for improving physical function (*p* = 0.056) and enhancing the quality of life in older people with chronic insomnia [47]. Quality of life is a key indicator in cognitive impairment research, as it reflects not only cognitive function but also emotional well-being, autonomy, and social interaction. Its assessment is essential to determine whether cognitive improvements translate into meaningful functional benefits. However, the lack of a standardized approach and a clear justification for its inclusion limits comparability between studies and the clinical relevance of the results.

### 4.2. Cognitive Function

There were no significant differences between EG and CG. A meta-analysis could not be performed due to the heterogeneity of the guidelines used. Within the studies [36,37,39,41,42], it is evident that the multimodal lifestyle intervention and the Tai Chi Qigong intervention did not significantly improve cognitive functions; however, interventions such as CBT, relaxation training and moderate-intensity aerobic exercise did obtain positive effects in various areas of cognitive functions studies have shown that multimodal interventions combining training and cognitive stimulation significantly improve cognitive function in individuals with cognitive deficits (*p* = 0.005) [48]. Similarly, a meta-analysis of Tai Chi revealed notable improvements in cognitive function, particularly in executive functions (*p* = 0.004), following 10 weeks to 1 year of training in individuals with cognitive impairment [49]. These findings highlight the importance of intervention dosage in achieving long-term cognitive benefits.

Additionally, an RCT demonstrated that a digital CBT intervention significantly reduced self-reported cognitive impairment by the end of the program, with these effects persisting for 6 months (*p* < 0.001) [50]. Moreover, another study evaluating mindfulness interventions over 12 months (*p* = 0.002) and progressive muscular relaxation over 18 months (*p* = 0.003) reported significant cognitive function improvements in individuals with cognitive deficits [51]. Consistent with other research on aerobic exercise, studies suggest that physical activity is a critical factor in cognitive improvement among individuals with mild cognitive impairment (MCI). An active lifestyle positively influences cognitive capacity (*p* = 0.015), potentially delaying or preventing the progression of MCI during aging [52]. The choice of cognitive assessment tools, influenced by education level, impacts result interpretation. The MMSE is less sensitive than the MoCA in detecting MCI, particularly in highly educated individuals. Lack of justification for tool selection can introduce biases, underscoring the need for appropriate interventions to support cognitive function in older people with MCI.

### 4.3. Sleep Quality

The meta-analysis reported significant improvements in sleep quality as measured by the PSQI. Multimodal lifestyle interventions, cognitive behavioral therapy, self-administered hypnosis, self-relaxation training, Tai Chi Qigong, moderate-intensity aerobic training, and physical activity programs [42,43] demonstrated significant improvements in sleep quality [36,37,38,39,40,41]. Consistent with other studies, a multimodal intervention that includes sleep hygiene education has positively affected sleep quality in older people (*p* = 0.007) [53]. Similarly, a recent meta-analysis of 10 RCTs demonstrated that CBT for insomnia significantly improves sleep quality (*p* < 0.001) by effectively altering behaviors and thought patterns related to sleep [54]. Furthermore, a meta-analysis found that hypnotherapy significantly reduced sleep latency (*p* = 0.01). However, these findings should be interpreted cautiously due to the small number of included studies and their methodological limitations [55]. Additionally, interventions combining meditation and gentle movement appear particularly beneficial for sleep quality. For example, a meta-analysis confirmed that Tai Chi Qigong has statistically significant effects on sleep quality (*p* < 0.00001) in older people, suggesting it is an effective intervention for those with sleep disorders [56]. Lastly, numerous studies on physical activity highlight its positive impact on sleep quality (*p* < 0.001), noting that exercise facilitates sleep onset and continuity by regulating circadian rhythms and promoting melatonin production [57]. Collectively, these findings indicate that non-pharmacological interventions are effective in significantly enhancing sleep quality among older adults with MCI.

### 4.4. Strengths and Limitations

Limitations of this systematic review with meta-analysis include (i) differences in the studies concerning the types of intervention and the dose applied, presenting difficulties in generalizing the results; (ii) the lack of evaluations related to the variables of quality of life and cognitive function in some studies, which affects the analysis of the results due to the lack of data; and (iii) the limitation in the number of available studies applied to older people with MCI, which affects the generalization of results and obtaining conclusions in this population. Its strengths are the following: (i) The incorporation of articles with different intervention approaches, which allows for a more specific analysis of the effectiveness of non-pharmacological treatments. (ii) Most of the included studies reporting significant improvements in the variables, but only non-pharmacological interventions were found to be an effective method for improving sleep quality in older people with MCI. (iii) The vast majority of studies evaluated sleep quality through standardized measures (PSQI), which may allow comparison of results across studies and interventions. By analyzing the reported results, further research is suggested to explore and investigate aspects of sleep quality, cognitive function, and quality of life. This would allow for more effective and evidence-based interventions for sleep disorders in older people with MCI.

### 4.5. Practical Applications

This systematic review with meta-analysis examines the efficacy of interventions for sleep disorders in older individuals with MCI, focusing on sleep quality, cognitive function, and quality of life. While interventions significantly improved sleep quality, not all showed notable benefits in cognitive function [36,37,41] or quality of life [36]. These findings highlight the need for individualized intervention protocols. Future research should optimize intervention doses and include long-term follow-ups to assess sustained effects. Cognitive behavioral therapy, aerobic exercise, and relaxation techniques notably improve sleep, while multimodal programs have limited effects on cognitive function and quality of life. Combining physical activity, cognitive stimulation, and relaxation strategies may enhance efficacy. Non-pharmacological interventions offer practical opportunities to improve quality of life and cognitive function. Integrating moderate-to-vigorous physical activity into community and residential settings, adapting CBT-I to digital formats, and incorporating practices like Tai Chi and relaxation training can improve sleep and mental well-being. Training caregivers to assist in these interventions can optimize outcomes, promoting sustained engagement and adherence.

## 5. Conclusions

Interventions for sleep disorders in older adults with MCI significantly improve sleep quality, as assessed by the PSQI. However, individual results showed limited effects on cognitive function and quality-of-life assessments. The evidence suggests that incorporating physical activity, cognitive stimulation, cognitive behavioral therapy, and relaxation strategies may enhance intervention efficacy. Nonetheless, further studies are required to establish optimal intervention designs, including the appropriate dose, duration, and specific components needed to achieve consistent improvements in quality of life, cognitive function, and sleep quality among older people with MCI. Additionally, future research should focus on personalized interventions that consider individual needs and preferences and explore the long-term effects of these interventions. This approach could lead to more effective and sustainable strategies for managing sleep disorders in this population.

## Figures and Tables

**Table 1:** Selection criteria used in the systematic review.

Criteria	Inclusion Criteria	Exclusion Criteria
Population	Studies were carried out with a population of mean aged 60 years and older diagnosed with MCI and with some sleep disorders.	Studies where the primary pathology differs from MCI and is carried out with a population of mean under 60 years.
Intervention	Studies containing non-pharmacological interventions for sleep disorders in older people with MCI with programs from 4 weeks onwards.	Studies whose focus of intervention was not related to non-pharmacological intervention programs in older people with MCI.
Comparison	Interventions with an EG focused on sleep treatment.	Lack of data and/or monitoring.
Outcomes	Studies that provide results on quality of life, cognitive function, and sleep quality through validated assessment tools.	No assessment of sleep quality is provided.
Study design	Randomized controlled trials.	Non-randomized, cross-sectional, retrospective, and prospective controlled studies.

EG: experimental group; MCI: mild cognitive impairment.

**Table 2:** Characteristics of the included studies.

Study	Country or Multicenter	Study Design	Sample	Groups (n)	Mean Age (Years)	Type of Intervention and Control Group	Training Volume			Training Intensity	Quality of Life (Assessment)	Cognitive Function (Assessment)	Sleep Quality (Assessments)	Main Outcomes
							Weeks	Frequency (Sessions /Week)	Session Duration (Minutes)					
[36]	Multicenter	RCT	Participants diagnosed with MCI	EG: 48 CG: 48	73.52 years old	Multimodal lifestyle intervention vs. Education + Attentional Control	24	1	120	Moderate	MVPA	ADAS-Cog Plus	PSQI, MW8 for objectively measured sleep efficiency	Multimodal lifestyle intervention versus education + attentional control: Both groups: ↔ There are no significant differences in cognitive function at 12 weeks (*p* = 0.684) and 24 weeks (*p* = 0.349). EG: ↔ There are no significant differences in %MVPA at 12 weeks (*p* = 0.776) and 24 weeks (*p* = 0.977). ↔ Changes in sleep efficiency at 24 weeks (*p* = 0.282). Changes in sleep quality: ↑ There is a significant difference between groups at 24 weeks (*p* = 0.040).
[37]	USA	RCT	Older adults diagnosed with MCI	EG: 14 CG: 13	89.3 years old	CBT-I vs. Nutrition class (Active control)	6	1	60	NR	SF-36 Physical Health, SF-36 Mental Health	HVLT-R, MOCA	ISI, AHI	CBT-I vs. Active Control: Both groups: ↔ No significant change in cognitive function measures (MOCA/HVLT-R) (*p* = 0.91/0.62). VR Arm: ↑ Significant improvement in sleep outcomes (ISI, sleep efficiency) (*p* < 0.001). VR Arm: ↑ Positive trend in executive functioning tasks compared to CG (*p* < 0.08). Both groups: ↔ A difference is identified only in the SF-36 Physical quality of life rating (*p* = 0.11).
[38]	USA	RCT	Adults with MCI	EG: 11 CG: 12	71.95 years old	Self-administered hypnosis intervention vs. White noise audio recordings	5	7	15	NR	NR	NR	PSQI, ESS	Self-Hypnosis vs. White: Noise Control Hypnosis Arm: ↑ Significant improvement in sleep outcomes (PSQI) (*p* < 0.001). Hypnosis Arm: ↑ Daytime sleepiness improved significantly from baseline to endpoint (ESS) (*p* = 0.044).
[39]	CN	RCT	Older people with reduced sleep quality	EG: 40 CG: 40	68.59 years old	Self-relaxation training (including Progressive Muscle Relaxation and meditation) vs. Sleep hygiene education	48	1	90	Moderate	NR	MMSE	PSQI, ESS	Self-Relaxation Training Versus Sleep Hygiene Education: EG: ↑ Significant improvements in sleep quality measured by the Pittsburgh Sleep Quality Index (*p* < 0.001). ↑ Significant improvements in cognitive function scores over time (*p* < 0.001).
[40]	Multicenter	RCT	Veterans aged 60 and older with insomnia symptoms	EG1: 52 EG2: 54 CG: 53	72.1 years old	CBT-I vs. General Sleep Education Control	5	1	60–90	NR	NR	NR	PSQI, ISI, Sleep efficiency according to actigraphy	CBT-I versus CG: INT Group: ↑ Significant improvement in sleep quality (PSQI) after treatment (*p* < 0.05), at 6 months (*p* < 0.05) and at 12 months (*p* < 0.05). EG: ↓ Significant reductions in Insomnia Severity Index (ISI) scores at all follow-up points (*p* = 0.002). ↔ There were no significant differences in sleep efficiency according to actigraphy at the different evaluation points (*p* = 0.27).
[41]	CN	RCT	Older adults with cognitive impairment	EG: 27 CG: 25	80.6 years old	Tai Chi Qigong vs. CG (standard care)	8	2	60	Low specification	Short-form12 Health Survey (SF-12v2)	Mini-Mental State Examination (MMSE) Memory inventory for Chinese questionnaire (MIC)	Chinese Pittsburgh Sleep Quality Index (CPSQI)	Tai Chi Qigong group vs. CG Both group: ↔ No significant differences in the SF-12 physical component, MMSE score, or MIC score (*p* > 0.05). Tai Chi Qigong group: ↑ Significant improvement in the SF-12 mental health component score at 2 months (*p* < 0.001). Changes in sleep quality: ↑Significant improvement in the CPSQI global score in the TCQ group at 6 months (*p* = 0.004).
[42]	CN	RCT	Participants diagnosed with MCI	EG: 60 CG: 60	75.78 years old	Moderate-intensity aerobic exercise training vs. General education program	16	3	60	Moderate	QOL-AD-C	MOCA	PSQI	EG: ↑Significant improvement in cognitive function (*p* < 0.001) compared to the CG. EG: ↑ Significant improvement in quality of life (*p* < 0.001) compared to the CG. EG: Changes in sleep quality: ↑ The treatment group had a significant improvement in sleep quality compared to the CG (*p* < 0.001).
[43]	TR	RCT	Older adults diagnosed with MCI	EG: 30 CG: 30	72.24 years old	Physical Activity Program vs. No participation in the physical activity program	20	4	80	Moderate	NR	SMMSE	PSQI	20-week Physical Activity Program vs. CG: EG: ↑ Significant improvement in sleep quality (PSQI scores) post-intervention (*p* < 0.05). ↑ Significant improvement in cognitive function (SMMSE scores) post-intervention compared to CG (*p* = 0.001).

ADAS-CogPlus: Alzheimer’s Disease Assessment Scale-Cognitive Plus; AHI: Apnea–Hypopnea Index; CBT-I: cognitive behavioral therapy for insomnia; CG: control group; CN: China; EG: experimental group; ESS: Epworth Sleepiness Scale; HVLT-R: Hopkins Verbal Learning Test-Revised; ISI: Insomnia Severity Index; MMSE: Mini-Mental State Examination; MCI: mild cognitive impairment; MOCA: Montreal Clinical Assessment; MVPA: moderate-to-vigorous physical activity; MW8: MotionWatch8©; NR: not reported; RCT: randomized controlled trial; PSQI: Pittsburgh Sleep Quality Index; QOL-AD-C: Quality of Life in Alzheimer’s Disease; SMMSE: Standardized Mini Mental State Examination; TR: Turkey; USA: United States of America. ↑: Significant increase/improvement in EG compared to the CG; ↓: Significant decrease in EG compared to the CG; ↔ There are no significant differences between groups.

**Table 3:** Assessment of methodological quality using the GRADEpro tool.

Certainty of Evidence							No of Patients		Effect		Certainty	Importance
No of Studies	Study Design	Risk Assessment	Inconsistency	Indirect Evidence	Vagueness	Other Considerations	[In Sleep Disorders]	[Conventional Treatment]	Relative (95% CI)	Absolute (95% CI)		
**Effect of a Multimodal Lifestyle Intervention on Sleep and Cognitive Function in Older Adults with Probable Mild Cognitive Impairment and Poor Sleep: A Randomized Clinical Trial**												
1	RCT	It is not serious	It is not serious	It is not serious	It is not serious	None	48/98 (49.0%)	50/98 (51.0%)	1.18 (−0.99 to 3.34)	88 more per 1000 (from 975 less to 1000 more)	++++ High	IMPORTANT
**Neuropsychological Functioning in Older Adults with Mild Cognitive Impairment and Insomnia Randomized to CBT-I or Control Group**												
1	RCT	It is not serious	It is not serious	It is not serious	It is not serious	None	14/28 (50.0%)	14/28 (50.0%)	Not estimable		++++ High	IMPORTANT
**Hypnosis Intervention for Sleep Disturbances in Individuals with Mild Cognitive Impairment: A Randomized Pilot Study**												
1	RCT	Serious	It is not serious	It is not serious	It is not serious	None	11/23 (47.8%)	12/23 (52.2%)	Not estimable		+++ Moderate	IMPORTANT
**Self-relaxation training can improve sleep quality and cognitive functions in the older: a one-year randomized controlled trial**												
1	RCT	It is not serious	It is not serious	It is not serious	It is not serious	None	40/80 (50.0%)	40/80 (50.0%)	Not estimable		++++ High	IMPORTANT
**Cognitive Behavioral Therapy for Insomnia in Older Veterans Using Nonclinical Sleep Coaches: Randomized Controlled Trial**												
1	RCT	It is not serious	It is not serious	It is not serious	It is not serious	None	106/159 (66.7%)	53/159 (33.3%)	6.5 (5.8 to −7.2)	1000 more by 1000 (from 1000 less to 1000 more)	++++ High	IMPORTANT
**Tai chi qigong as a means to improve night-time sleep quality among older adults with cognitive impairment: a pilot randomized controlled trial**												
1	RCT	Very serious	It is not serious	It is not serious	Serious	None	27/52 (51.9%)	25/52 (48.1%)	−2.67 (−4.51 to −0.83)	1000 less by 1000 (from 1000 less to 880 less)	+ Very Low	IMPORTANT
**Effects of a moderate-intensity aerobic exercise program on the cognitive function and quality of life of community-dwelling elderly people with mild cognitive impairment: A randomized controlled trial**												
1	RCT	It is not serious	It is not serious	It is not serious	It is not serious	None	60/120 (50.0%)	60/120 (50.0%)	−1.257 (−1.609 to −0.825)	1000 less by 1000 (from 1000 less to 913 less)	++++ High	IMPORTANT
**Effects of Physical Activity Program on cognitive function and sleep quality in elderly with mild cognitive impairment: A randomized controlled trial**												
1	RCT	Very serious	It is not serious	It is not serious	Serious	None	30/60 (50.0%)	30/60 (50.0%)	Not estimable		+ Very Low	IMPORTANT
